# Mill pond sediments as the indicator of the environment of the drainage area (an example of Liswarta River, Odra basin, Poland)

**DOI:** 10.1007/s11356-017-0909-y

**Published:** 2017-12-12

**Authors:** Maria Fajer, Martyna Anna Rzetala

**Affiliations:** Faculty of Earth Sciences, University of Silesia, Bedzinska 60, 41-200 Sosnowiec, Poland

**Keywords:** Trace elements, Metals, Bottom sediments, Land use, Agricultural lands, Channel-type reservoirs, Liswarta river, Mill-dam

## Abstract

The geochemical characteristics of sediments deposited within a channel-type reservoir situated behind the weir of a water mill on the River Liswarta (southern Poland) were studied in relation to land use in the catchment. The catchment in question is an agricultural one with large forest areas. The contamination of sediments with trace elements was assessed using the geoaccumulation index. The sediments studied were moderately to heavily contaminated with As, Cu, Co, Ni and Ba. They were also heavily contaminated with Sr. Additionally, V and Cr contamination ranged from heavy to extreme. The basic composition of sediments and the trace elements present in them indicate both natural and anthropogenic sources of pollution. Mill impoundments provide zones where the sediments transported by rivers accumulate. Within the Liswarta catchment, their removal may cause the remobilisation of contaminated alluvial deposits.

## Introduction

Land use has a significant impact on surface waters (e.g. Jagus and Rzetala 2011; Rzetala et al. 2014; Calijuri et al. 2015), on the processes occurring on river valley floors (e.g. Kondolf et al. 2002; Merritts et al. 2011; Quiñonero-Rubio et al. 2014), and on the geochemical characteristics of alluvial sediments (e.g. Niemitz et al. 2012). In the past, human-induced environmental transformations in river valleys were primarily related to agricultural land use. Currently, many river valleys are subject to strong multi-directional human pressure (e.g. Balpande et al. 1996; Brannstrom and Oliveira 2000; Kessler and Stroosnijder 2006; Gutierrez et al. 2009; Fu et al. 2010; Jagus and Rzetala 2012; Meshesha et al. 2012; Rzetala and Jagus 2012; Machowski et al. 2012; Rzetala et al. 2013a, b, 2015).

From the Middle Ages onward, the major structures constructed in river channels in agricultural catchments have included mill impoundments, mostly involving small ponds. Water mills have exerted a significant influence on the geomorphology of river channels and on the processes occurring on flood plains and therefore they have attracted the researchers’ interest for many years (e.g. Foster and Walling 1994; Trimble 1998; Downward and Skinner 2005; Pizzuto and O'Neal 2009; Schenk and Hupp 2009). Mill ponds have also played a significant role in the accumulation of sediments and are even considered the major factor in historical sedimentation (Walter and Merritts 2008; Merritts et al. 2011; Wegmann et al. 2012). Bottom sediments in these ponds are often used as indicators of human pressure (e.g. Walter and Merritts 2008; Schenk and Hupp 2009; Merritts et al. 2011; Niemitz et al. 2012; James 2013).

Many water mills had channel-type reservoirs resulting from increasing the water level in the river channel. Although the efficiency of those reservoirs in catching the sediments transported by the river was limited, in many agricultural catchments they still form the few “geoarchives” available.

Bottom sediments in anthropogenic water bodies and alluvia are good indicators of environmental features and of the changes taking place in the catchment because they are the main depositories of the pollutants supplied to the rivers (Rzetala et al. 2013a, b; Rzetala 2015a, b, c). The analysis of the geochemical spectrum of the sediments deposited in water bodies reflects the impact of land use and of non-agricultural sources of pollution on the catchment (Nascimento and Mozeto 2008; Rendina and Fabrizio de Iorio 2012).

The Liswarta has a catchment covered by agricultural land and forests. Just as on many other European rivers, dozens of water mills with impoundments and mill ponds operated on the Liswarta and its tributaries from the Middle Ages until the mid-twentieth century. Hydraulic energy was used by both grain and industrial mills (hammer mills, saw mills). After the mills had been closed down, a significant proportion of the dams and weirs were destroyed and the still existing ones, after being repaired, are now used for other purposes including for small hydropower plants.

The purpose of the study was the evaluation of the impact of land use in an agricultural and forest catchment on the geochemical characteristics of the sediments deposited in a small channel-type reservoir of the former Marcelin water mill in Krzepice.

## Study area

**Fig. 1:**
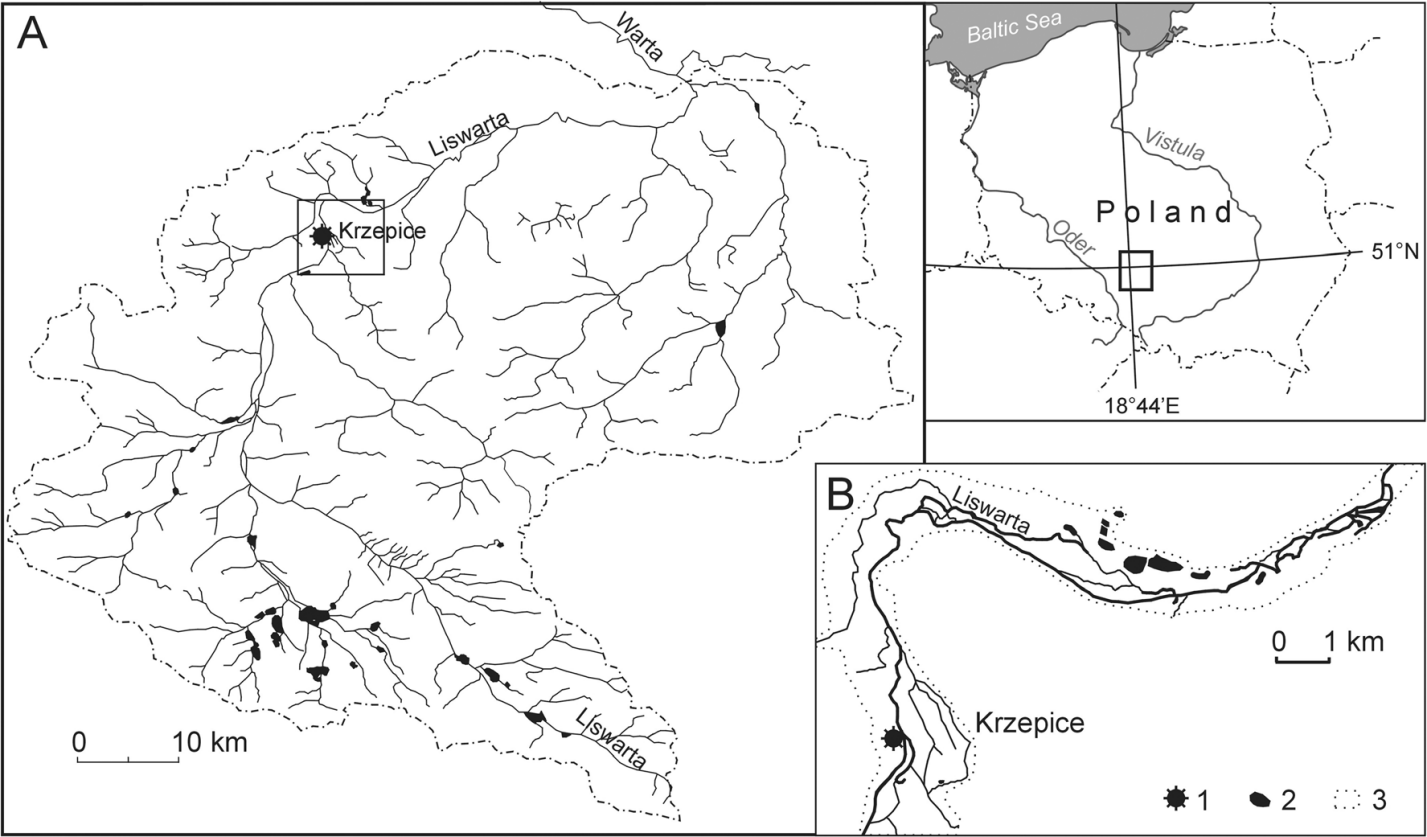
Location of study area. **a** Catchment of the River Liswarta, **b** location of the Marcelin water mill: 1 – Marcelin water mill, 2 – pond, 3 – floodplain

The Liswarta is a left bank tributary of the upper River Warta (River Oder basin) with a length of 93 km. Its catchment, which has an area of 1557.7 km^2^, is located in the western part of the Polish Uplands. The Liswarta catchment is asymmetrical with the right-bank part accounting for about 67% of its area (Fig. 1).

**Fig. 2:**
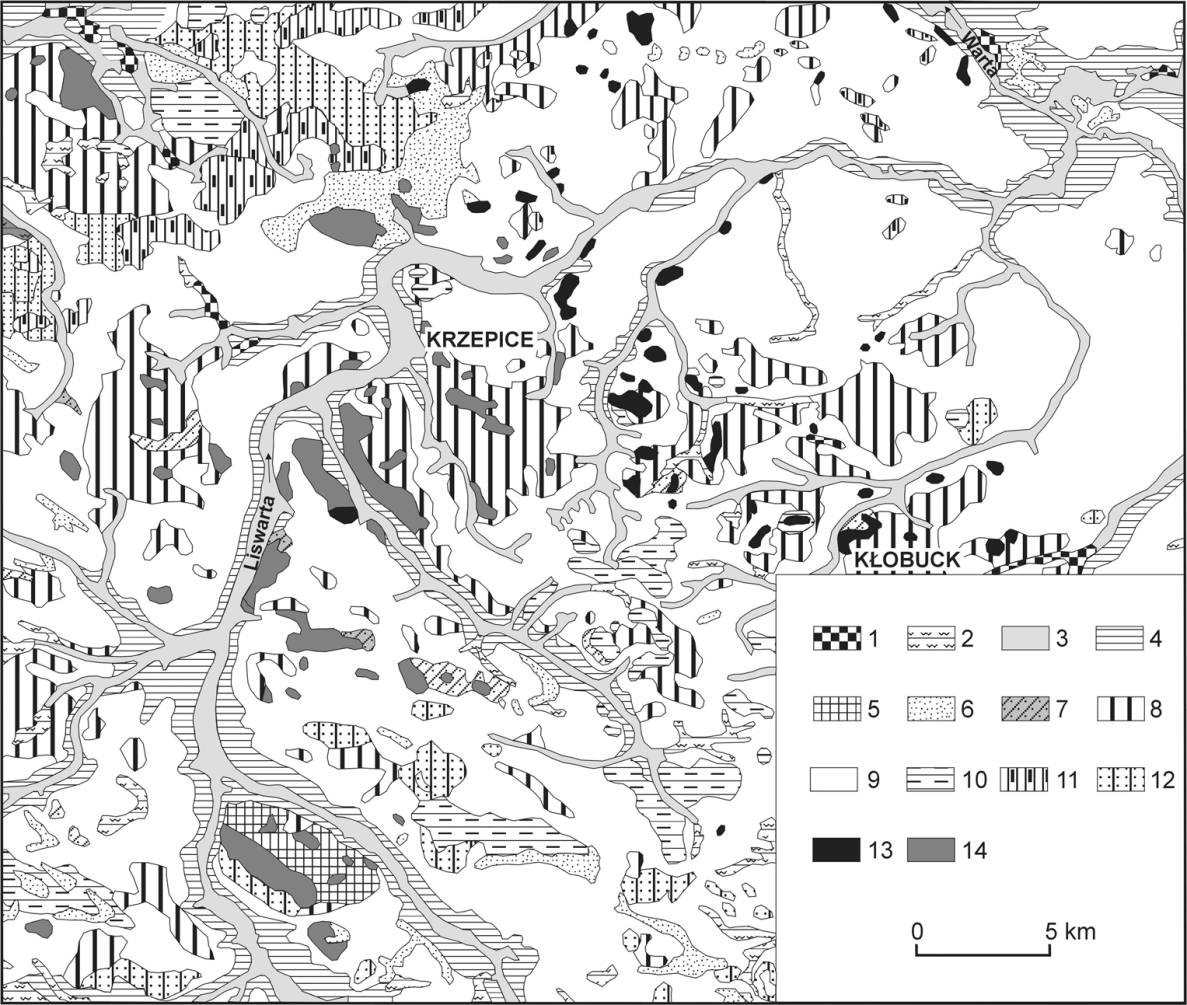
Quaternary formations in the basin of the middle and lower Liswarta (based on Fajer 2004, simplified). 1 – peats; 2 – mud; 3 – fluvial silts, sands and gravels (Holocene valley bottoms); 4 – fluvial silts, sands and gravels (floodplain terraces); 5 – weathered clay, loam and sand; 6 – aeolian sands; 7 – deluvial sand and loam; 8 – glacial tills; 9 – fluvioglacial sands and gravels; 10 – clays, silts, sands and gravels (kame and kame terraces); 11 – sands, gravels and boulders (moraines); 12 – glacial sands, gravels and boulders; 13 – outcrops of older substrate (Upper Jurassic limestones); 14 – outcrops of older substrate (Triassic and Lower and Middle Jurassic sands, sandstones, conglomerates, clays and mudstones)

This area is covered by quaternary deposits of varying thickness—glacial tills, sands, fluvioglacial and fluvial gravels, silts and clays (Fig. 2). The solid geology consists of Jurassic rocks. In the upper and middle parts of the catchment, there are sandstones, sands, clay shales, clays and mudstones with siderite iron ores. Iron ore was mined in the catchment from the fourteenth century, and mining on an industrial scale started in the twentieth century and lasted until the early 1980s. The lower part of the catchment consists of limestones (Osika 1987).

The area examined is characterised by old glacial landforms with signs of structural denudation. There are zones of extensive denudation troughs used by rivers and structural thresholds with gorges.

The density of the river network varies from 1.5–2.0 km/km^2^ in the upper part of the catchment to 0.45 km/km^2^ in its lower part. This results from the heterogeneous geological structure of the catchment area. In the lower part of the catchment, water infiltrates more easily into the karst limestone rocks of the substrate which are covered with a thin layer of Quaternary sediments. The average gradient of the Liswarta river channel is 1.22‰ (Fajer et al. 2012).

The climate in the study area is moderate with an average annual temperature of 7–8 °C, average July temperature of 18–19 °C and average January temperature of 3 °C. Average annual precipitation totals 580 to 680 mm. The vegetation period lasts from 210 to 220 days (Kondracki 1981).

The Liswarta and its tributaries have even hydrological regimes, which include a spring freshet (usually in March) and minor floods in the summer (July–August). The share of underground sources in the total annual flow is 55–65%. The reduced flow period lasts from May to November with a minimum in September (Dynowska 1971). Annual fluctuations in water stages and flows are insignificant. The multiannual average amplitude of water stages in the Liswarta ranges from 120 to 140 cm. The average flow in the upper reach amounts to 1.50 m^3^/s, and in the lower reach it is 6.89 m^3^/s. The average high flow rate is 40.5 m^3^/s. Statistically, floods in the Liswarta River catchment occur every 3–3.5 years. In the twentieth and twenty-first centuries, the greatest floods were recorded in 1924, 1939, 1947, 1953, 1977, 1982, 1987, 1997 and 2010. During the catastrophic flood of 1997, a flow rate of 116 m^3^/s was recorded. There are fish ponds and small water bodies used for recreational purposes in the upper part of the catchment (Fajer et al. 2012; Czaja et al. 2014).

The predominant soils in the catchment area are luvisols, podzols and cambisols originating from sandy and loamy formations. In the river valleys, organic histosols are present (bog and less commonly peaty soils). In the middle reach of the Liswarta valley, fluvisols (alluvial soils) dominate. In terms of their agricultural suitability, the soils in the Liswarta catchment are of medium and low quality (Soil and Agricultural Map 1987). In the upper and middle parts of the catchment, a significant portion of the soils (50–70%) are acidic or very acidic (Pasieczna 2003).

Agriculture (arable land and permanent grassland) is the dominant land use in the Liswarta catchment. It accounts for the largest share in the middle part of the catchment, e.g. 75–85% in the vicinity of Krzepice (source: Regional Water Management Board [Regionalny Zarząd Gospodarki Wodnej] in Poznań). As concerns the crop structure, cereals predominate covering about 84% of the area of arable land (Regional Data Bank 2005). Meadows in the valley floors were intensively used for agricultural purposes until the end of the 1980s. Currently, they are used extensively or not at all. Forests account for 34.5% of the total catchment area of the Liswarta; in the upper part of the catchment forest density is very high, reaching up to 76–81% locally.

Population density in the area analysed ranges from 93 to 96 persons/km^2^. The population mostly lives in rural areas (about 77%). Larger towns include Kłobuck—13,085 residents and Krzepice—4524 residents (Regional Data Bank 2013).

Presently, the waters of the Liswarta River and of its tributaries are not significantly polluted with metals. The water is polluted with biogenic compounds associated with agricultural activity and household sewage. A high concentration of NO_3_ was recorded in the Liswarta River. In its tributaries, high concentrations of NH_4_, NO_2_, NO_3_ and total nitrogen were recorded. In one of the tributaries (the Biała Oksza River), the permissible total phosphorus concentration of 0.45 g/dm^3^ was exceeded (Matysik et al. 2015; www.katowice.pios.gov.pl).

## Materials and methods

### Data on land use change

Changes in land use between 1897 and 1998 were analysed using archival maps supplemented by information from historical sources. A map of land use in the Liswarta catchment in the nineteenth century was drawn up on the basis of archival topographic maps. The 1:100,000 scale *Karte des Westlichen Russlands* (sheets published from 1870 to 1919) and the 1:100,000 scale *Mapa Taktyczna Polski* issued by the Military Geographical Institute (Wojskowy Instytut Geograficzny–WIG) from 1919 to 1939 (based on German editions from 1918, topographic survey carried out in 1897) were used. The 1:25,000 scale *Topographische Karte* published from 1870 to 1945 and the 1:25,000 scale *Mapa Szczegółowa Polski* published by WIG from 1929 to 1939 (topographic survey carried out from 1881 to 1889) were also used.

For the analysis of land use in the Liswarta catchment in 1998, the 1:50,000 scale *Mapa Topograficzna Polski* produced in the National System of Geodetic Coordinates 1992 was used (topographic survey carried out from 1991 to 1993).

Four basic categories of land use were distinguished: agricultural land including arable land and permanent grassland, forests, developed areas both urban and rural, and water bodies. The remaining areas included wasteland and watercourses.

The aforementioned maps were also used to produce a map presenting the distribution and changes in the number of water mills in the Liswarta catchment from 1897 to 1998. To this end 1:10,000 scale topographic maps, which were produced on the 1965 system of coordinates (topographic survey carried out in 1990), were also used.

The data obtained from the topographic maps were supplemented with the results of the interpretation of aerial photographs. To this end, aerial photographs from the years 1955 and 1956 were used as well as a digital orthophotomap made available by the Centre for Surveying and Map Documentation (Centralny Ośrodek Dokumentacji Geodezyjnej i Kartograficznej). The analysis of the orthophotomap made it possible to refine the data on the current distribution of mill impoundments or their remnants.

In order to determine the construction dates of mills in the reach of the Liswarta analysed, historical sources and archival plans concerning the development of the town of Krzepice, which were obtained from the National Archive in Częstochowa, were used in addition to historical literature.

### The Marcelin water mill in Krzepice—a case study

The Marcelin water mill in Krzepice lies on the middle reach of the Liswarta and the sediments deposited in the mill impoundment zone were selected for geochemical tests (Fig. 1).

**Fig. 3:**
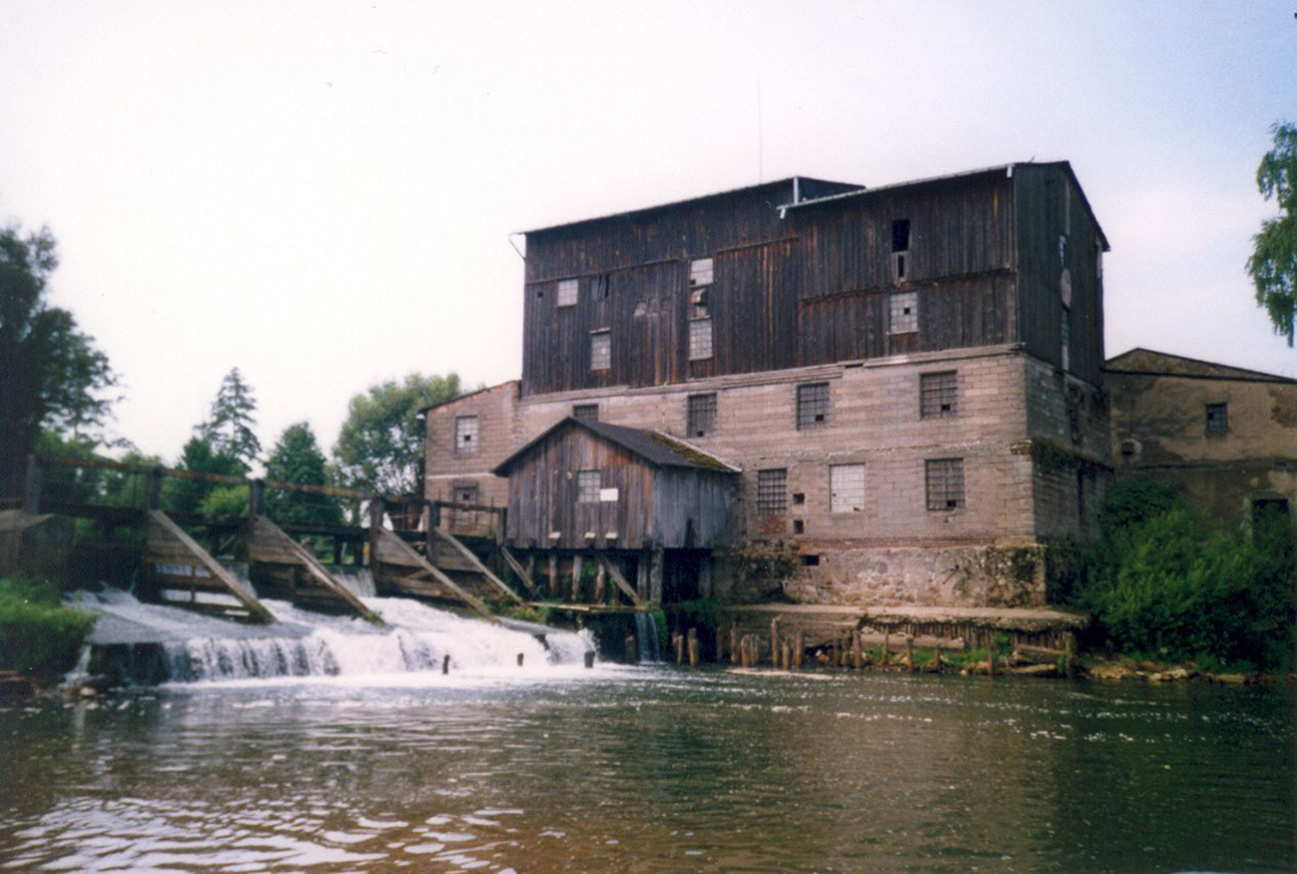
Marcelin water mill (as at 2003)

The mill (Fig. 3) was constructed in 1876. By 1909, it already operated as a water-motor mill powered by a 30 hp. water turbine and it also had a 15 hp. water wheel (www.wystawa1909.pl). The extant structure of the mill was built in 1930. A wooden weir was constructed next to the mill forming a small channel-type reservoir. Currently, the mill is closed and the weir was rebuilt in 2008.

The mill was located on a river branch or on an enlarged former mill race of an older, defunct mill that was located ca. 1 km upstream from the Marcelin mill.

### Field mapping

Field mapping was performed in the section of the Liswarta river channel and of the floodplain in the area of Krzepice using a 1:10,000 scale topographic map. The basic structural and textural characteristics of the sediments deposited in the channel-type reservoir above the mill weir were identified. The thickness of accumulated sediments was determined. A cross-section of the channel was drawn showing the succession of sediments; drawings and photographs were made. A total of 40 samples were collected from the floodplain alluvia near Krzepice in order to measure their pH using the potentiometric method.

### Sampling of bottom sediments

Samples of the bottom sediments were collected from the channel-type reservoir near the Marcelin water mill in Krzepice in order to study grain size distribution and conduct geochemical analyses. Sediment samples were collected in March 2008 in a dry river channel during weir reconstruction. The sediments were collected manually into polyethylene bags directly from the profile of the alluvia deposited at the bottom of the channel-type reservoir. A mixed sample was prepared, which was representative of the top silt layer (Marcelin 1 sample), and also another mixed sample, which consisted of the silt present 0.5 m below (Marcelin 2 sample).

### Laboratory analyses

An analysis of bottom sediment grain size distribution was conducted using the sieving and areometric methods at the Laboratory for Soil and Rock Analysis of the Faculty of Earth Sciences of the University of Silesia in Sosnowiec (Poland). Sandy sediments were analysed using the dry sieving method according to the relevant standards (Loveland and Whalley 2000; ISO 11277: 2009). In the analysis, a set of calibrated analytical sieves with circular meshes (0.1, 0.25, 0.5, 1.0, 2.0 and 5.0 mm) was used. The samples were agitated for 10 min. The determination of the content of the < 0.1-mm fraction was conducted using Bouyoucos’s areometric method as modified by Casagrande and Prószyński (Ryzak et al. 2009). Pretreatment consisted of boiling the samples for 30 min with the addition of 1.5 g anhydrous sodium carbonate to disperse the sediment. Percentage shares of individual fractions were determined by measuring the density of the suspension at strictly defined time intervals at a temperature of 20 °C. The Prószyński areometer and sedimentation tables were used, which enabled the direct determination of the percentage of suspended particles at the time of measurement.

Sediment samples were prepared for geochemical tests at the Laboratory for Soil and Rock Analysis of the Faculty of Earth Sciences of the University of Silesia in Sosnowiec (Poland). The basic preparation of samples for geochemical analyses consisted of grinding the material in a mortar. The < 0.063-mm fraction, which is useful for geochemical tests, was isolated using chemically inert sieves and a vortex mixer.

The chemical composition of bottom sediments was analysed at the accredited ACTLABS (Activation Laboratories Ltd.) laboratory in Ancaster, Canada in accordance with the standards observed at that laboratory (www.actlabs.com).

The basic components: SiO_2_, Al_2_O_3_, Fe_2_O_3_, MnO, MgO, CaO, Na_2_O, K_2_O, TiO_2_, P_2_O_5_ and loss on ignition as well as Ba, Be, Sr, V, Y and Zr, were determined using inductively coupled plasma (ICP) atomic emission spectrometry. The samples were prepared and analysed using the Thermo Jarrell-Ash ENVIRO ICP II sequential analyser system (Ancaster, Canada). Samples are prepared and analysed in a batch system. Each batch contains a method reagent blank, certified reference material and 17% replicates. Samples are mixed with a flux of lithium metaborate and lithium tetraborate and fused in an induction furnace. The molten melt is immediately poured into a solution of 5% nitric acid containing an internal standard, and mixed continuously until completely dissolved (~30 min) (www.actlabs.com). The same method was used to measure Cu, Pb, Zn, Ni, Cd and S after a complete dissolution of samples (www.actlabs.com). A 0.25-g sample dry aliquot is digested with a mixture of HClO_4_, HNO_3_, HCl and HF at 200 °C until fumed and is then diluted with *aqua regia*. For the ICP analysis, reagent blanks with and without the lithium borate flux are analysed, as well as the method reagent blank. Interference correction verification standards are analysed. Calibration is performed using multiple USGS and CANMET certified reference materials. Two of the standards are used during the analysis for every group of ten samples. This standard brackets the group of samples. The sample solution is also spiked with internal standards and is further diluted and introduced into a Perkin-Elmer SCIEX ELAN 6000 ICP/MS (Ancaster, Canada) using a proprietary sample introduction methodology (www.actlabs.com).

As, Br, Ce, Co, Cr, Cs, Eu, Hf, La, Nd, Rb, Sc, Sb, Sm, Th and U were determined by instrumental neutron activation analysis (INAA) on 1 g aliquots. A 1 g aliquot is encapsulated in a polyethylene vial and irradiated with flux wires and an internal standard (1 for 11 samples) at a thermal neutron flux of 7 × 10^12^ n cm^−2^ s^−1^. After a 7-day period, to allow Na-24 to decay, the samples are counted on a high purity Ge detector with resolution of better than 1.7 KeV for the 1332 KeV Co-60 photopeak. Using the flux wires, the decay-corrected activities are compared to a calibration developed from multiple certified international reference materials. The standard present is only a check on accuracy and is not used for calibration purposes. From 10 to 30% of the samples are rechecked by re-measurement (www.actlabs.com).

Mercury content was determined by atomic absorption spectrometry (AAS) using the cold vapour technique for 0.5 g dry aliquots (www.actlabs.com). Hg analysis is performed on a Perkin-Elmer FIMS 100 cold vapour Hg analyser (Ancaster, Canada). A 0.5 g sample is digested with aqua regia at 90 °C. The Hg in the resulting solution is oxidised to the stable divalent form. Since the concentration of Hg is determined via the absorption of light at 253.7 nm by Hg vapour, Hg (II) is reduced to the volatile free atomic state using stannous chloride. Argon is bubbled through the mixture of sample and reductant solutions to liberate and to transport the Hg atoms into an absorption cell. The cell is placed in the light path of an atomic absorption spectrophotometer. The maximum amount absorbed (peak height) is directly proportional to the concentration of mercury atoms in the light path. Measurement can be performed manually or automatically using a flow injection technique (FIMS).

The contamination of sediments with trace elements was assessed using the geoaccumulation index (Eq. 1) developed by G. Müller (Förstner and Müller 1981).

1 $$ {I}_{geo}={\log}_2\frac{C_n}{1.5{B}_n} $$

where:

I_geo_: geoaccumulation index;
C_n_: concentration of the element in question in bottom sediments;
B_n_: geochemical background for the element in question;
1.5: coefficient expressing natural variation in the content of the element in question in the environment.

The geoaccumulation index (Eq. 1) distinguishes several classes of sediment quality depending on the concentration of substances considered to be pollutants (Förstner and Müller 1981):

- I_geo_ ≤ 0.0—practically uncontaminated,
- 0.0 < I_geo_ < 1.0—uncontaminated to moderately contaminated,
- 1.0 < I_geo_ < 2.0—moderately contaminated,
- 2.0 < I_geo_ < 3.0—moderately to heavily contaminated,
- 3.0 < I_geo_ < 4.0—heavily contaminated,
- 4.0 < I_geo_ < 5.0—heavily to extremely contaminated,
- I_geo_ > 5.0—extremely contaminated.

To determine the degree of contamination of sediments in the Liswarta using the geoaccumulation index, the following geochemical background values for sediments in the river were adopted as determined by Lis and Pasieczna 1995, which (calculated as geometric means for the individual elements) are: 5.0 mg/kg—As, 50.0 mg/kg—Ba, 0.5 mg/kg—Be, 0.7 mg/kg—Cd, 3.0 mg/kg—Co, 4.0 mg/kg—Cr, 4.0 mg/kg—Cu, 6.0 mg/kg—Ni, 11.0 mg/kg—Pb, 0.157%—S, 6.0 mg/kg—Sr, 4.0 mg/kg—V and 103.0 mg/kg—Zn. The geochemical background value was determined using statistical methods on the basis of samples collected from inland water bodies and watercourses within a 5 × 5 km grid (Lis and Pasieczna 1995).

## Results

Farmland is the dominant land use in the Liswarta catchment. In period from 1897 to 1998 subject to analysis, the changes in land use that occurred were slight. The changes in land use structure affected 7.5% of the catchment area. These changes primarily concern the areas occupied by farmland and forests (Table 1). Until the early 1970s, traditional agriculture predominated in the study area and chemical fertilisers and pesticides were not used in large amounts. The 1970s and 1980s were a period when the use of fertilisers and plant protection products increased.

**Table 1:** Areas of individual land use categories in the Liswarta catchment in 1897 and 1998

Land use categories	1897		1998		Changes in land use from 1897 to 1998	
	km^2^	%	km^2^	%	km^2^	%
Farmland	986.30	63.32	940.46	60.38	45.84	− 2.94
Forests and plantations	480.41	30.84	537.83	34.53	57.42	+ 3.69
Developed areas both urban and rural	74.14	4.76	61.51	3.95	12.63	− 0.81
Water bodies	9.15	0.59	10.20	0.65	1.05	+ 0.06
Other	7.7	0.49	7.7	0.49	0.0	0.0

**Fig. 4:**
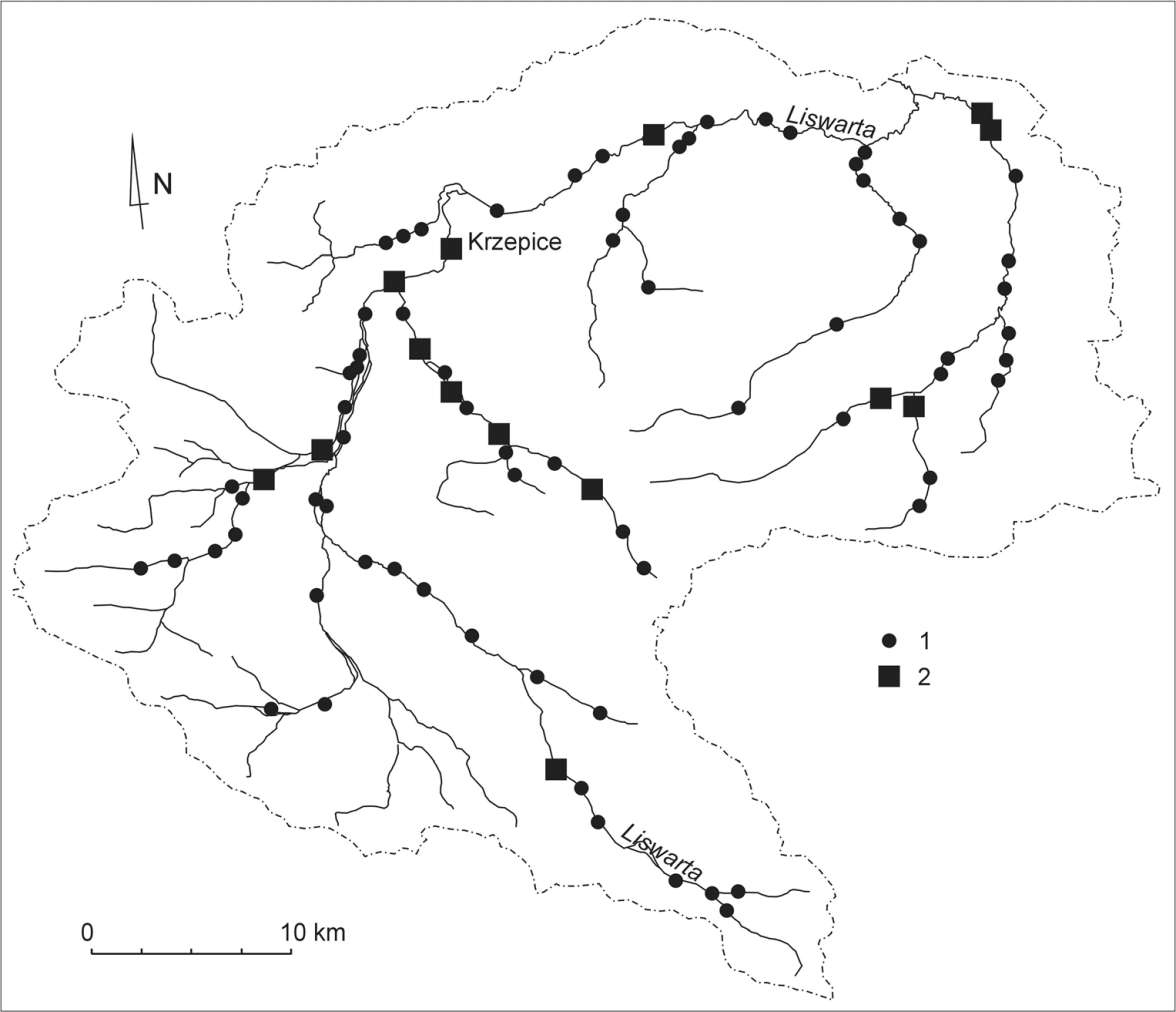
Location of water mills in the Liswarta catchment in 1897 and in 1998: 1 – mill impoundments present in 1897, 2 – mill impoundments present in 1998

Between 1897 and 1998, very significant changes occurred in the number of mill impoundments on the Liswarta and its tributaries. Most of the mills were closed down and their weirs were entirely or partially demolished. Out of the 83 mill impoundments that functioned in 1897, only 14 remained in 1998 (Fig. 4). Of those, four mill impoundments are on the Liswarta, including the Marcelin mill in Krzepice, and the remaining ten are situated on its tributaries.

**Fig. 5:**
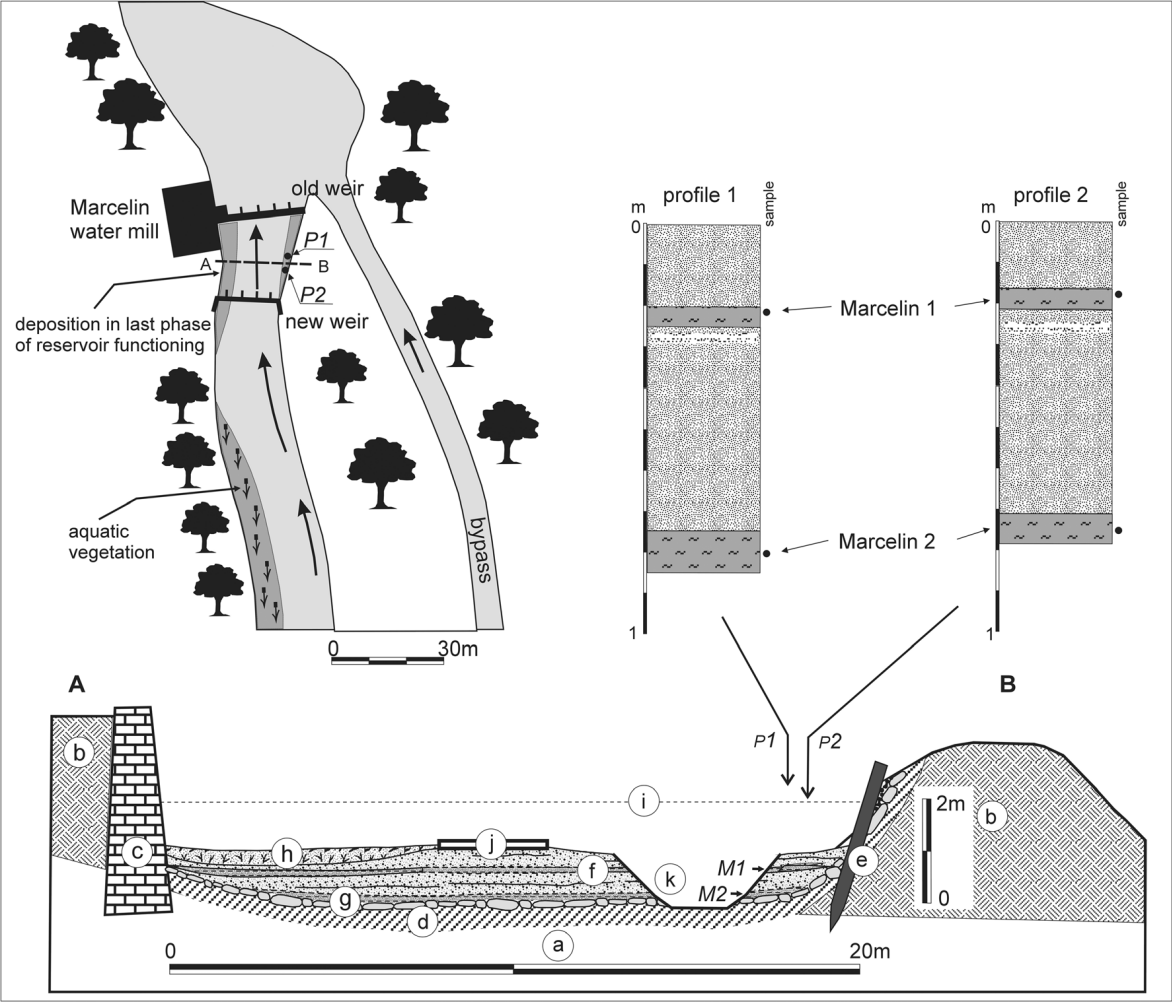
A plan and cross-section of the channel-type reservoir of the Marcelin water mill (as at 2008). Substrate and hydro-engineering structures: (a) older alluvia; (b) embankments; (c) retaining wall; (d) clay sealing reinforced with limestone boulders and Scandinavian erratic boulders in the top layer; (e) palisade with fascine. Sediments filling the channel-type reservoir: (f) sands of various grain sizes, locally with silt laminae; (g) silts with an admixture of sand; (h) silt deposits with coarse vegetable detritus and garbage filling the inlet into the water turbine chamber; (i) water level; arrows indicate levels at which samples were collected; (j) concrete access road (associated with the new weir construction phase); (k) drainage ditch (associated with the new weir construction phase)

Prior to the 2008 reconstruction of the reservoir, its bottom above the weir had been sealed with clay and protected from erosion by weir. At the bottom of the reservoir, coarse- and medium-grained sands and silts were deposited (Fig. 5). The sediments probably started to accumulate in the years 1900–1909 after the mill had been modified and the water wheel replaced with the Francis turbine. The sediment sequence started with a layer of grey silt around 5–10 cm thick. The silt layer, which was observed in multiple locations, was probably present along the entire width of the channel. These silts exhibited little variation caused by the admixture of other fractions (mean grain size in phi units: 4.17–5.54). Higher, a layer of coarse- and medium-grained sands up to 50 cm thick was present, which included thin silt laminae (mean grain size: 0.07–2.38). Above them, there was a layer of grey silt with a thickness of around 5 cm, which was observed along the entire width of the channel (mean grain size: 4.37–4.9). It has also been preserved above the new weir where it is covered by a brown detritus level at many locations. Above the silt layer examined, 20–30-cm thick sands, sometimes including fine gravel (mean grain size: 1.03–2.37) accumulated in the entire reservoir. During the operation of the mill, the water current was directed towards the main sluice on the left side of the dam, and therefore the thickness of sediments was smaller there at the time.

After the water turbine had been decommissioned in the years 1955–1956, the current was directed towards the sluices in the middle. Ultimately, the most recent silt sediments including coarse plant detritus and garbage (bottles, polystyrene, etc.) accumulated within the turbine channel zone.

The deposition of fine-grained silt sediments occurred under calm sedimentation conditions associated with slowly flowing or stagnant water—this could have happened when water supply to the reservoir was shut off due to failures, downtime or repairs. At these times, the water was directed to the bypass.

The grain size distribution of the sediments deposited above the mill impoundment results from the characteristics of the surface formations present in the Liswarta catchment. Sandy and loamy sediments prevail in the area. The Liswarta carries little material in the form of suspension.

The basic composition of sediments was dominated by silica (60.46–60.70%), Al_2_O_3_ (18.30–18.85%) and organic matter. Loss on ignition ranged from 10.13 to 10.30% (Table 2). The shares of the following oxides were much lower: Fe_2_O_3_ (4.36–5.68%), K_2_O (2.58–2.72%), MgO (1.09–1.19%) and TiO_2_ (1.03–1.04%). The following oxides accounted for the lowest shares of the chemical composition (at tenths of a percent): CaO (0.51–0.57%), Na_2_O (0.18–0.19%), P_2_O_5_ (0.11%) and MnO (0.03%).

**Table 2:** Basic chemical composition of sediments in the channel-type reservoir of the Marcelin Mill

Component	Unit	The lower limit of detection	Marcelin 1 sample	Marcelin 2 sample
SiO_2_	%	0.01	60.46	60.70
Al_2_O_3_	%	0.01	18.30	18.85
Fe_2_O_3_	%	0.01	4.36	5.68
MnO	%	0.01	0.03	0.03
MgO	%	0.01	1.19	1.09
CaO	%	0.01	0.57	0.51
Na_2_O	%	0.01	0.18	0.19
K_2_O	%	0.01	2.72	2.58
TiO_2_	%	0.005	1.03	1.04
P_2_O_5_	%	0.01	0.11	0.11
Loss of ignition	%	0.01	10.30	10.13

Among the elements identified in the sediments of the Liswarta, the following exhibited the highest concentrations (Table 3): Ba (380–404 mg/kg), Zr (249–266 mg/kg), V (145–150 mg/kg), Rb (130–170 mg/kg) and Cr (122–132 mg/kg). The following elements were present in the sediments studied at levels from a dozen to several dozen mg/kg: Ce, Zn, Sr, Nd, Ni, La, Cu, Y, Pb, Th, As, Co, Sc. Concentrations of the aforementioned elements (except for Zr and Sr) exceed the levels recorded in the upper continental crust layer and in European river sediments. The following elements were present at up to a few mg/kg: Hf, Cs, Sm, U, Yb, Be, Eu, Sb. Lu was present at a few tenths of a milligram per kilogram at most. The average sulphur content determined was 0.022–0.119%. Mercury was present at 49–56 μg/kg.

**Table 3:** Trace elements in the bottom sediments of the channel-type reservoir of the Marcelin mill

Component	Unit	The lower limit of detection	Upper continental crust*	Europe stream sediment median**	Marcelin 1 sample	Marcelin 2 sample
Ba	mg/kg	3.0	550.0	306.0	404.0	380.0
Zr	mg/kg	2.0	190.0	589.0	249.0	266.0
V	mg/kg	5.0	60.0	23.0	145.0	150.0
Rb	mg/kg	20.0	112.0	44.0	170.0	130.0
Cr	mg/kg	1.0	35.0	46.5	132.0	122.0
Ce	mg/kg	3.0	64.0	40.6	94.0	93.0
Zn	mg/kg	1.0	71.0	42.5	93.0	87.0
Sr	mg/kg	2.0	350.0	79.0	78.0	77.0
Nd	mg/kg	5.0	26.0	16.8	52.0	59.0
Ni	mg/kg	1.0	20.0	9.0	64.0	52.0
La	mg/kg	0.2	30.0	20.0	49.7	51.7
Cu	mg/kg	1.0	25.0	9.0	21.0	27.0
Y	mg/kg	1.0	22.0	15.8	26.0	25.0
Pb	mg/kg	5.0	20.0	15.0	25.0	22.0
Th	mg/kg	0.5	10.7	7.0	15.4	16.8
As	mg/kg	2.0	1.5	4.0	17.0	16.0
Co	mg/kg	1.0	10.0	3.0	20.0	15.0
Sc	mg/kg	0.1	11.0	16.3	14.2	14.7
Hf	mg/kg	0.5	5.8	11.4	8.3	9.2
Cs	mg/kg	0.5	3.7	< 4.0	10.1	7.8
Sm	mg/kg	0.1	4.5	3.2	6.0	5.8
U	mg/kg	0.5	2.8	1.0	5.1	4.5
Yb	mg/kg	0.1	2.2	1.88	3.3	3.4
Be	mg/kg	1.0	3.0	0.73	3.0	3.0
Eu	mg/kg	0.1	0.88	0.55	1.1	1.1
Sb	mg/kg	0.2	0.2	0.4	1.1	0.7
Lu	mg/kg	0.05	0.32	0.3	0.36	0.36
S	%	0.001	No data	0.510	0.119	0.022
Hg	μg/kg	5.0	No data	31.0	49.0	56.0
Au	μg/kg	5.0	1.8	No data	< 5.0	< 5.0
Ir	μg/kg	5.0	0.02	No data	< 5.0	< 5.0
Se	mg/kg	3.0	50.0	1.5	< 3.0	< 3.0
W	mg/kg	3.0	2.0	0.72	< 3.0	< 3.0
Bi	mg/kg	2.0	0.127	0.08	< 2.0	< 2.0
Mo	mg/kg	2.0	1.5	0.47	< 2.0	< 2.0
Br	mg/kg	1.0	No data	No data	< 1.0	< 1.0
Ta	mg/kg	1.0	2.2	0.73	< 1.0	< 1.0
Ag	mg/kg	0.5	0.05	0.22	< 0.5	< 0.5
Cd	mg/kg	0.5	0.098	0.31	< 0.5	< 0.5
Tb	mg/kg	0.5	0.64	0.45	< 0.5	< 0.5

*Data after Taylor and McLennan (1995)

**Data after De Vos et al. (2006)

Around a dozen elements were present in amounts below the lower limit of detection of the methods used: Au and Ir (< 5.0 μg/kg), Se and W (< 3.0 mg/kg), Bi and Mo (< 2.0 mg/kg), Br and Ta (< 1.0 mg/kg) and Ag, Cd and Tb (< 0.5 mg/kg).

## Discussion

### Changes in land use

Land use patterns persist for a long time in the study area. Land use in the Liswarta catchment has not changed significantly since the late nineteenth century, and it remains an agricultural and forest area. Agriculture has always played an important role in the economy of the area.

The slight decrease in farmland area and increase in the share of forests that were recorded in the period analysed are a result of the poor quality of the soil which is often earmarked for afforestation.

Although forms of land use have not changed, farmland cultivation techniques have evolved. In subsequent years, however, the amount of mineral fertilisers used in agriculture significantly decreased. Currently, the amount of nitrogen has halved and the amounts of phosphorus and potassium are just a quarter of those used from 1974 to 1989 (Statistical Yearbook 1974, 2014).

Major changes took place in the river valleys of the Liswarta catchment during the period analysed. As a result of the closing down of water mills, the number of mill impoundments that had existed for decades or even centuries decreased dramatically. The analysis of archival maps indicates the stability of some of the water mill locations. Given the large number of water mills that operated in the Liswarta River catchment at the end of the nineteenth century, their overall impact was significant. Mill ponds increased surface retention and their presence indirectly raised groundwater levels around the ponds and also above dams. This effect was most visible in the small valleys of Liswarta River tributaries, among others in the Pankówka River valley (around a dozen kilometres from the Marcelin mill) where in the nineteenth century seven ponds were located 0.5–1.2 km apart. Around each of these ponds, the groundwater level in the valley was very shallow. The ground was waterlogged up to 0.7 km above the pond, which was recorded in the structure of soil profiles (Fajer 2003). Between 1897 and 1998, 83% of water mills in the Liswarta River catchment were decommissioned. The groundwater level dropped. It is difficult, however, to unequivocally determine the effect of the removal of water mill dams on the fall in groundwater levels, since this process overlapped with the large-scale drainage works conducted from 1950 to 1980 in order to drain the waterlogged meadows in the river valley. These works accelerated the outflow of water from the catchment and lowered the groundwater table by 0.6 m on average (Fajer 2003).

The trend towards the closing down of water mills was a global one due to technological changes in the milling industry and also to economic and political factors (Downward and Skinner 2005).

### Impact of land use in the catchment on alluvial geochemistry

Pollution load is often reflected in the chemical composition of alluvial deposits and of bottom sediments in mill ponds. These provide an important indicator in the study of environmental pollution owing to the fact that concentrations of contaminants in such sediments are much higher than in water. Metal pollution of the aquatic environment is one of the most important indicators of environmental change caused by human activity (Alloway and Ayres 1997). Fe and Mn oxides and hydroxides, organic matter and clay minerals play a major role in the retention of metals in aquatic sediments (Kabata-Pendias and Pendias 1999).

In the sediments accumulated above mill impoundments, we should expect pollutants that may indicate the types of human pressure present in the Liswarta catchment during the last century (agriculture, iron ore mining, transport routes).

During the period of the nineteenth and twentieth centuries analysed, there were no industrial facilities that would supply pollutants to the Liswarta in the immediate vicinity of the Marcelin mill. However, we know from historical sources that a smithy operated on the banks of the Liswarta in the sixteenth and seventeenth centuries, probably from 0.5 to 1 km upstream from the Marcelin mill.

The chemical composition of the sediments examined is conditioned by the geological substrate, weathering and soil formation processes, the nature of the debris and also by patterns of land use in the catchment (Kabata-Pendias and Pendias 1970; Tiller 1989). This concerns both the macro- and microelements present. In the case of some components, it may point to human impact on the environment.

The dominant component of the sediments deposited in the Marcelin mill impoundment zone is SiO_2_. A high loss on ignition indicates a significant organic matter content in the sediments. This is allochthonous matter. In addition to quartz/silica and organic matter, basic components also include Al_2_O_3_, Fe_2_O_3_ and oxides of the following elements: Mn, Mg, Ca, Na, K, Ti and P.

The high content of Al_2_O_3_ in the sediments (> 18%) largely reflects their clay mineral content. Soils are a significant source of Al which leaches out as a result of acidification. Most soils in the Liswarta catchment are acidic soils formed on sands under coniferous forests, while farmland has been acidified by the introduction of ammonium nitrate fertilisers. Water in the Liswarta and its tributaries has a pH in the range from 7.0 to 7.7 and the concentration of Al^+3^ ions is low at up to 102 μg/l (www.katowice.pios.gov.pl). Tests of the pH of alluvial deposits on the Liswarta floodplain near Krzepice demonstrated that at depths from 0 to 20 cm, pH is in the 4.03–6.21 range while alluvial samples collected from a depth of 40–60 cm had pH values in the 4.96–6.77 range. Fe_2_O_3_ content is slightly above average while MnO content is low. According to De Vos et al. (2006), Fe_2_O_3_ content in European river sediments ranges from 0.11 to 18.3% with an average value of 4.07%. The higher concentration of Fe_2_O_3_ in the Liswarta catchment may be a consequence of the presence of siderite iron ores in the central part of the catchment as well as small bog iron deposits in floodplain alluvia. The share of MgO, CaO, Na_2_O and P_2_O_5_ is low in the sediments studied. This may stem from the easy solubility and high mobility of Mg and Ca compounds. Na content in the sediments of most Polish rivers is low (< 0.5%) (De Vos et al. 2006). The slightly increased K_2_O content in the sediments of the Liswarta may be the result of the adsorption of this component by clay minerals and organic matter and may also be related to the use of potash fertilisers. The high TiO_2_ content in Liswarta sediments should be noted. TiO_2_ has low mobility in the environment and is transported in a colloidal state. Anthropogenic Ti anomalies are rarely found (De Vos et al. 2006). In the Liswarta catchment, the supply of TiO_2_ to sediments is most likely related to the historical iron smelting industry that was based on siderite ores.

Apart from macroelements, trace elements were also found in the sediments studied. Some trace elements (e.g. Zn, Cu) are considered necessary for organisms to thrive, while others are considered unnecessary and even harmful such as Pb, Cd, Cr and Ni (Kabata-Pendias and Pendias 1999).

Geoaccumulation index values (*I*_geo_) indicate a variation in sediment quality in the Liswarta in terms of concentrations of the elements analysed (Table 4). The concentrations of S, Cd, Zn and Hg in the sediments studied do not amount to contamination. With respect to Pb concentrations, the sediments range from uncontaminated to moderately contaminated. Given that the concentration of Pb does not exceed 50 mg/kg, the sediments examined can be classified as uncontaminated (Lis and Pasieczna 1995; Bojakowska and Sokołowska 1998; Rzetala et al. 2013a, b). As concerns the As, Cu, Co, Ni and Ba content, the sediments studied belonged to the next two classes (moderately and moderately to heavily contaminated). The sediments examined were heavily contaminated with Sr, and their V and Cr contamination ranged from heavy to extreme.

**Table 4:** Geoaccumulation index (*I*_geo_) values and sediment quality classes

Component	Geoaccumulation index (*I*_geo_) value		Sediment class	
	Marcelin 1 sample	Marcelin 2 sample	Marcelin 1 sample	Marcelin 2 sample
S	− 0.98	− 3.42	*I*_geo_ ≤ 0.0	*I*_geo_ ≤ 0.0
Cd	− 1.07	− 1.07	*I*_geo_ ≤ 0.0	*I*_geo_ ≤ 0.0
Ba	2.43	2.34	2.0 < *I*_geo_ < 3.0	2.0 < *I*_geo_ < 3.0
V	4.59	4.64	4.0 < *I*_geo_ < 5.0	4.0 < *I*_geo_ < 5.0
Cr	4.46	4.35	4.0 < *I*_geo_ < 5.0	4.0 < *I*_geo_ < 5.0
Zn	− 0.73	− 0.83	*I*_geo_ ≤ 0.0	*I*_geo_ ≤ 0.0
Sr	3.12	3.10	3.0 < *I*_geo_ < 4.0	3.0 < *I*_geo_ < 4.0
Hg	− 0.61	− 0.42	*I*_geo_ ≤ 0.0	*I*_geo_ ≤ 0.0
Ni	2.83	2.53	2.0 < *I*_geo_ < 3.0	2.0 < *I*_geo_ < 3.0
Cu	1.81	2.17	1.0 < *I*_geo_ < 2.0	2.0 < *I*_geo_ < 3.0
Pb	0.60	0.42	0.0 < *I*_geo_ < 1.0	0.0 < *I*_geo_ < 1.0
As	1.18	1.09	1.0 < *I*_geo_ < 2.0	1.0 < *I*_geo_ < 2.0
Co	2.15	1.74	2.0 < *I*_geo_ < 3.0	1.0 < *I*_geo_ < 2.0

The heightened concentrations of Cr, V, Sr, Ba and Co are related to the geological structure of the catchment area and the exploitation of siderite iron ores. Research by Razowska (2001) shows that Middle Jurassic ore-bearing clays include many minerals containing Fe, Pb, Zn, Ba, Sr, Mn, Mg and other elements; as a result of weathering (oxidation and dissolution), these minerals are a source of macro- and microelements. The concentration of Sr in the alluvia is associated with the weathering of carbonate rocks or the erosion of calcium-rich soils. Strontium easily migrates to aquatic environments during weathering processes. Naturally increased amounts of Ba are characteristic of argillaceous rocks (Lis and Pasieczna 1995; Pasieczna 2003). High concentrations of Ba and V may originate from the slags that were generated during the smelting of iron ore in the former iron works situated on the Liswarta and its tributaries (the Pankówka and Łomnica), which operated until the 1920s. During studies conducted in the Liswarta catchment, it was recorded that metallurgical slag was used to improve dirt roads and to reinforce dikes and even the banks of the river itself.

Other human-made pollutants may also constitute sources of Cr, V and Sr. Coal ash contains large quantities of V (more than 1000 mg/kg) and Ba (1274 mg/kg) (Kabata-Pendias and Pendias 1999; Lis and Pasieczna 1995). Coal is the primary material used for heating in household heaters and small boilers in the Liswarta catchment. Solid coal combustion products in the form of ash are often deposited on the slopes of river terraces and even in some oxbow lakes and old, inactive mill races. In rural areas, this is a significant threat. The fact that alluvia and soil are obviously contaminated with Cr is the result of the activities of the metallurgical and tanning industries. The extraction and processing of iron ores (initially bog iron and limonite ores and later siderite ones) in the Liswarta catchment started in the Middle Ages, and began on an industrial scale in the twentieth century continuing until the 1980s. Similarly, the long-standing activities of tanneries have resulted in water pollution (Pawlikowski et al. 2006; Molik et al. 2004; Armienta et al. 2001). The sewage and waste produced by tanneries may contain more than 1% Cr (Bojakowska 1994). A tannery situated on the upper reach of the Liswarta, about 40 km upstream from the Marcelin Mill, operated from 1924 until the late 1970s. This plant was a major source of Cr pollution. Owing to its low chemical mobility, Cr is transported mechanically to locations at a considerable distance from the source areas (Lis and Pasieczna 1995). The concentration of Cr in alluvia in the Liswarta is an order of magnitude higher than that in the River Kłodnica (Barbusiński and Nocoń 2011), which flows through the urban areas of Upper Silesia. It even exceeds the levels recorded in the bottom sediments of anthropogenic water bodies in heavily urbanised and industrialised areas (Rzetala et al. 2013a, b). A source of Sr in agricultural areas may be runoff from soil that has been fertilised with lime, which is enriched with Sr (Lis and Pasieczna 1995).

A review of the literature demonstrates (Franciskovic-Bilinski 2006; Zeng and Wu 2013) that elevated levels of Ba in Liswarta sediments can also be associated with the supply of industrial and municipal wastewater.

Increased As concentrations may have a natural origin as well as providing evidence of contamination (Masson et al. 2009; Barringer et al. 2011; Liu et al. 2014; Rzetala 2015).

Copper can be both geogenic and anthropogenic (including, inter alia, plant protection products used in agriculture) (Zeng and Wu 2013; El Bouraie et al. 2010).

The elevated Ni content in Liswarta sediments is probably related to the extraction of iron ores. Increased concentrations of this element are found in regions where metallic ores are present (Alexakis and Gamvroula 2014; O'Neill et al. 2015). In the vicinity of Częstochowa, the highest concentrations of Ni have been found in soils around the waste dumps left after the extraction of iron ore (Pasieczna 2003).

Only the content of some metals can be compared with the results obtained in the studies concerning other rivers in agricultural areas. The concentrations of Ni, Zn, Pb and Cu in the Liswarta near the Marcelin water mill are similar to those in bottom sediments of small flow-through reservoirs in the upper part of the neighbouring catchment of the River Prosna (Gałka and Wiatkowski 2010a, b), which is also agricultural, as well as to those in water bodies in the Małopolska, Podkarpacie and Opole regions (Gałka and Wiatkowski 2010b). However, those water bodies have catchments that are both smaller in size (approx. 10–14 km^2^) and much younger (they have only been present since 1986 and 1994) than is the case of the Marcelin mill; for the latter reason, no long-term accumulations of metal contamination have been found there. Lower concentrations of metals have been recorded in non-industrialised areas of Poland, including on the River Słupia in northern Poland (Banach and Chlost 2007) where a cascade of reservoirs forms a barrier to the migration of pollutants, and on the River Wieprz that flows through rural areas in eastern Poland (Kozieł and Zgłobicki 2010).

### Sedimentation efficiency in mill channel-type reservoirs

The construction of low dams and weirs in river channels at locations where water mills operated forced the deposition of sediments in the zones above them. If the volume of water exchanged is low, these zones serve as sedimentation tanks. However, in channel-type reservoirs with flow-through characteristics, such as the one at the Marcelin water mill, one should expect less contaminated sediments. The alluvia deposited above the weir of the Marcelin water mill are mainly supplied by transit. They accumulate the pollutants supplied from the upper and middle parts of the Liswarta catchment. The sediments deposited are not thick and range from 50 cm to approx. 85 cm. As opposed to natural flow-through water bodies, sedimentation processes in the channel-type reservoir of the Marcelin mill could have been affected by the operation of the mill and the associated regulation of the water flow regime, including periods when the reservoir was not used at all.

Studies performed on English and American rivers demonstrate that most water mills had channel-type reservoirs that exhibited low sedimentation efficiency (Downward and Skinner 2005; Trimble 1998). However, studies conducted in the mid-Atlantic region of the USA indicate that low mill dams located on small rivers are highly efficient (40–80%) in trapping the sediments transported (Merritts et al. 2011). These authors point out that above mill impoundments, sandy deposits were sedimented that contained metallurgical slag, charcoal and clay material, which indicates an industrial land use in the eighteenth and nineteenth centuries. The studies conducted by Niemitz et al. (2012) demonstrate that deposits containing significant amounts of P and metals accumulate above old mill impoundments in agricultural areas. Interesting information was provided by Coulthard and Macklin (2003). More than 100 years after the disappearance of mining sources of pollution, 70% of the contaminated river sediments were still present in the catchment. The contamination of channel sediments with metals probably persists for several hundred years (Ciszewski 1999; Niemitz et al. 2012). These pollutants pose a significant threat to the environment.

The removal of a number of mill impoundments and the regulation of the channels of the Liswarta and some of its tributaries contribute to the intensification of erosion processes which may cause the remobilisation of polluted alluvia and their reincorporation into younger alluvia. In the Liswarta River channel, after the culmination of the flood, fine-grained sandy deposits and silts that originated from the upper reaches of the river were probably accumulated above mill dams, in places with lower gradients. The remobilisation of contaminated channel alluvia is significantly contributed to by extreme flooding episodes during which the metals trapped in the suspension may be transported over very long distances (Ciszewski 1999). However, some of the sediments were deposited on the floodplain. There are reasons to believe that phases of major sediment remobilisation were also associated with the regulation of the river channel in its upper reach. However, there are no data concerning the intensity of these processes. Nowadays, sediments in small channel-type reservoirs adjacent to old mills in the Liswarta River catchment may be remobilised during upgrades of hydraulic structures, e.g. when a small hydroelectric power plant is to be commissioned at the location where a mill used to operate.

On the other hand, the studies conducted in neighbouring catchments indicate that a significant proportion of pollutants are deposited in floodplain sediments, and these are remobilised most intensively in the reaches where the channel meanders. In the regulated river reach, pollutants are mostly transported from the channel to the floodplain (e.g. Macklin and Klimek 1992; Ciszewski and Malik 2004; Ciszewski et al. 2004), similarly as in the upper Liswarta River catchment. Similarly, the studies conducted in the USA (Schenk and Hupp 2009; Niemitz et al. 2012; Csiki and Rhoads 2010, 2014) and in the UK (Coulthard and Macklin 2003) demonstrate that the degradation or removal of thousands of old mill dams result in the remobilisation of considerable amounts of sediments contaminated with metals.

## Conclusions

1. The analysis of maps demonstrates that in the period from 1897 to 1998, agricultural and forest patterns of land use in the Liswarta catchment have not changed. There was a slight increase in the area of forest and plantation at the expense of the least valuable farmland. During the period analysed, 83% of water mills were closed down, as a result of which the number of dams and weirs in the river channels dropped precipitously.
2. The impoundments constructed for water mills provide zones for the accumulation of the sediments transported by rivers. Patterns of land use within the catchment in the last century have been recorded in the geochemical characteristics of the Liswarta sediments deposited within the impoundment zone of the Marcelin water mill in Krzepice.
3. Increased concentrations of TiO_2_ and elements such as As, Cu, Co, Ni, Ba, Sr, V, and Cr indicate the existence of several pollution sources, both with natural (geogenic, pedogenic) and anthropogenic origins. The main sources of pollution include areas where iron ores used to be extracted and smelted, former tanneries, the chemicals used in agriculture and household/municipal pollution.
